# Centers for Disease Control and Prevention Expert Panel Meetings on Prevention and Treatment of Anthrax in Adults

**DOI:** 10.3201/eid2002.130687

**Published:** 2014-02

**Authors:** Katherine A. Hendricks, Mary E. Wright, Sean V. Shadomy, John S. Bradley, Meredith G. Morrow, Andy T. Pavia, Ethan Rubinstein, Jon-Erik C. Holty, Nancy E. Messonnier, Theresa L. Smith, Nicki Pesik, Tracee A. Treadwell, William A. Bower, the Workgroup on Anthrax Clinical Guidelines

**Affiliations:** Centers for Disease Control and Prevention, Atlanta, Georgia, USA (K.A. Hendricks, S.V. Shadomy, M.G. Morrow, N.E. Messonier, T.L. Smith, N. Pesik, T.A. Treadwell, W.A. Bower);; National Institutes of Health, Bethesda, Maryland, USA (M.E. Wright);; University of California, San Diego School of Medicine, San Diego, California, USA (J.S. Bradley);; University of Utah, Salt Lake City, Utah, USA (A.T. Pavia);; University of Manitoba, Winnipeg, Manitoba, Canada (E. Rubenstein);; Stanford University, Stanford, California, USA (J.-E. Holty)

**Keywords:** anthrax, Bacillus anthracis, bacteria, bioterrorism and preparedness, antitoxin, raxibacumab, expert panel meeting, prevention, treatment, adults, Centers for Disease Control and Prevention

## Abstract

The Centers for Disease Control and Prevention convened panels of anthrax experts to review and update guidelines for anthrax postexposure prophylaxis and treatment. The panels included civilian and military anthrax experts and clinicians with experience treating anthrax patients. Specialties represented included internal medicine, pediatrics, obstetrics, infectious disease, emergency medicine, critical care, pulmonology, hematology, and nephrology. Panelists discussed recent patients with systemic anthrax; reviews of published, unpublished, and proprietary data regarding antimicrobial drugs and anthrax antitoxins; and critical care measures of potential benefit to patients with anthrax. This article updates antimicrobial postexposure prophylaxis and antimicrobial and antitoxin treatment options and describes potentially beneficial critical care measures for persons with anthrax, including clinical procedures for infected nonpregnant adults. Changes from previous guidelines include an expanded discussion of critical care and clinical procedures and additional antimicrobial choices, including preferred antimicrobial drug treatment for possible anthrax meningitis.

Anthrax has been recognized as an infectious disease of animals and humans for millennia. Within the United States, animal anthrax is reported in most years, but naturally occurring human anthrax is rare. Worldwide, however, the disease is common in wild and domestic animals and not uncommon among persons who interact with animals in agricultural regions of South and Central America, sub-Saharan Africa, central and southwestern Asia, and southern and eastern Europe (*1*). Biodefense experts often place *Bacillus anthracis* at or near the top of the list for potential threat agents. Inhalation anthrax is particularly deadly, as demonstrated by the 1979 accidental release of *B. anthracis* from a military microbiology facility in the Sverdlovsk region of Russia; 88% (66/75) of patients reported with inhalation anthrax died (*2*). More recently, humans have acquired disease from exposure to spores released purposefully as a bioterrorist weapon (*3*) and accidentally from naturally occurring sources (*4*,*5*).

## Methods

In early 2011, the Centers for Disease Control and Prevention (CDC) invited persons from CDC, the National Institutes of Health, the US Food and Drug Administration (FDA), and university medical centers with expertise in anthrax and critical care medicine to develop an agenda and a list of potential expert panelists to revise the current guidance for prevention and treatment of anthrax. In October 2011 and March 2012, CDC held subject matter–expert panel meetings to review available scientific information and update previous guidelines for prevention and treatment of anthrax. Meeting attendees (see end of article) included clinicians with experience caring for the most recently hospitalized patients with naturally occurring cases of inhalation, gastrointestinal, or injection anthrax and academic and federal civilian and military researchers with anthrax expertise. Specialties represented included internal medicine, pediatrics, obstetrics, infectious disease, emergency medicine, critical care, pulmonology, hematology, and nephrology.

At the meetings, attendees reviewed data from published literature on human anthrax case reports, human and animal clinical trials, anthrax prevention and treatment, and unpublished antitoxin efficacy and safety data. Workgroups were formed to address clinical management, antimicrobial drug use, and antitoxin use. The workgroups drafted changes to previous guidelines, vetted them before the expert panel, and reached general agreement. After the meeting, workgroup members further refined the proposed changes by teleconference and email exchanges. The final draft was distributed to all meeting participants for review and comment. This article summarizes the presentations and discussions of the meeting and updates previous CDC guidelines for prevention and treatment of anthrax in nonpregnant adults.

## Pathogenesis

*B. anthracis*, the causative agent of anthrax, is a nonmotile spore-forming, gram-positive, rod-shaped bacterium. The spores of *B. anthracis*, which can remain dormant in the environment for decades, are the infectious form, but vegetative *B. anthracis* rarely causes disease (*1*). Spores introduced through the skin lead to cutaneous or injection anthrax; those introduced through the gastrointestinal tract lead to gastrointestinal anthrax; and those introduced through the lungs lead to inhalation anthrax. After entering a human or animal, *B. anthracis* spores are believed to germinate locally or be transported by phagocytic cells to the lymphatics and regional lymph nodes, where they germinate; or both (*6*). *B. anthracis* begins producing toxins within hours of germination (*7*). Protective antigen (PA) and edema factor (EF) combine to form edema toxin (ET) and PA and lethal factor (LF) combine to form lethal toxin (LT). After binding to surface receptors, the PA portion of the complexes facilitates translocation of the toxins to the cytosol, in which EF and LF exert their toxic effects (*8*).

The toxins have several physiologic effects. In sedated and mechanically ventilated dogs challenged with 24-h LT infusions to simulate release during anthrax infection, the toxin produced gradual but progressive hypotension associated with reductions in systemic vascular resistance and left ventricular ejection fraction that persisted for ≤72 h (*9*). Similar challenges with ET produced rapid hypotension associated with sharp and persistent decreases in central venous pressure and systemic vascular resistance. Such changes suggest that both toxins alter the peripheral vasculature, but that LT might also have a direct effect on depressing myocardial function, and ET causes venous and arterial relaxation. Both toxins were associated with progressive renal and hepatic dysfunction because of direct toxic effects or secondarily to hypoperfusion (*9*).

Gross pathologic lesions observed in non-human primates (NHPs) used in aerosol challenge models of inhalation anthrax include edema, congestion, hemorrhage, and necrosis in the lungs and mediastinum. Splenitis and necrotizing or hemorrhagic lymphadenitis involving the mediastinal, tracheobronchial, and other lymph nodes are common (*10*). Primary pulmonary lesions, including those of pneumonia, are occasionally observed. Meningeal involvement ranging from edema, congestion, hemorrhage, and necrosis to suppurative or hemorrhagic meningitis, usually secondary to hematogenous spread from other types of anthrax, occurs in ≤77% of animals studied (*10*). Autopsy findings for persons who died from inhalation anthrax in Sverdlovsk and in the United States (*3*) are consistent with findings from the NHP studies. Persons who died had extensive amounts of serosanguinous fluid in pleural cavities and edema and hemorrhage of the mediastinum and surrounding soft tissues, and 48% had cerebral edema, 21% had ascites, 17% had pericardial effusions, and 14% had petechial rash. Mediastinal lymph nodes and spleen also showed hemorrhage and necrosis (*11*,*12*).

## Critical Care Measures and Procedures

### Evaluation

Early diagnosis of anthrax and initiation of appropriate treatment, particularly administration of a combination of antimicrobial drugs, are critical to improving survival. After treatment, uncomplicated cutaneous anthrax has a mortality rate of <2%. However, even with antimicrobial drug treatment and modern critical care, injection, gastrointestinal, and inhalation anthrax have mortality rates of 28% (*13*), ≥40% (*14*); and 45% (*3*,*15*), respectively. Anthrax meningitis is nearly always fatal, even with treatment (*16*). Many patients with cutaneous anthrax can be treated as outpatients. However, patients with symptoms or signs of systemic involvement (e.g., tachycardia, tachypnea, hypotension, hyperthermia, hypothermia, leukocytosis) or with lesions that involve the head, neck, or upper torso or that are large, bullous, multiple, or surrounded by edema, have higher mortality rates (*17*). Hospitalization is warranted for all patients with systemic cutaneous anthrax; gastrointestinal, injection, or inhalation anthrax; or anthrax meningitis or bacteremia.

Initial evaluation of patients suspected of having anthrax (Technical Appendix Table 1) should be similar to the standard evaluation for patients with an acute febrile illness and should have an emphasis on obtaining pretreatment blood and other appropriate cultures. However, failure to fulfill systemic inflammatory response syndrome criteria should not decrease concern for sepsis because patients with systemic anthrax might not initially appear critically ill. Inhalation anthrax can have a prodromal phase followed by a fulminant phase (*3*). Patients with systemic anthrax have had debilitating symptoms, followed first by transitory improvement and then by precipitous hemodynamic deterioration (*18*). Because of this potential for sudden decompensation, hospitalized patients should have careful hemodynamic monitoring, including continuous pulse oximetry and telemetry (Technical Appendix Table 1). Chest computed tomography might be needed to identify the characteristic widened mediastinum and pleural effusions as well as common that are inapparent on chest radiographs (e.g., pericardial effusion). Unless contraindicated, lumbar puncture should be performed to rule out meningitis.

### Hemodynamic Support

Despite potential pathophysiologic differences between *B. anthracis* septic shock and septic shock caused by other bacteria, standard sepsis and septic shock guidelines should be followed for anthraz patients, including guidelines for fluids, vasopressors, blood products, and invasive hemodynamic monitoring (*19*). Microangiopathic hemolytic anemia, coagulopathy, thrombocytopenia, and hemorrhage commonly occur with anthrax infections; these complications must be aggressively managed, and might pose contraindications to invasive central catheter placement. Fresh frozen plasma and plasmapheresis should be considered, and fibrinogen levels should be kept >100 mg/dL. Because of the risk for coagulopathy, mechanical rather than pharmacologic prophylaxis is preferred for prevention of deep vein thrombosis. An echocardiogram might be needed to identify pericardial effusions, as reported for 3 of 10 patients (postal workers) with inhalation anthrax in 2001 (*3*).

### Mechanical Ventilation

In addition to the need for mechanical ventilation for respiratory distress or airway protection for persons with altered mental status, some patients with anthrax might require respiratory support for airway edema. Substantial edema with fatal outcome can occur with cutaneous lesions involving the head, neck, or thorax, and with oropharyngeal lesions (*20*). Thus, aggressive monitoring for airway compromise is warranted in patients with such lesions.

In inhalation anthrax, although respiratory failure is more consistent with reaccumulating pleural effusions than with adult respiratory distress syndrome, standard mechanical ventilator principles apply (*21*). Airway pressures may be difficult to interpret if there is loss of pulmonary compliance from excessive chest wall edema or restriction by pleural or peritoneal effusions. The need for ventilation in some patients and the duration of ventilation in others may be reduced by pleural space drainage.

### Adjunctive Corticosteroids

There are no randomized trials of corticosteroid use for human anthrax and no animal data that support its use. However, several small observational studies of adjunctive corticosteroids for cutaneous anthrax of the head and neck appear to favor their use in such situations (*22*,*23*). Although there are also limited data on steroid use for documented anthrax meningitis, adjunctive intravenous dexamethasone is the standard of care for patients with suspected bacterial meningitis and should be started at the time of initial antimicrobial drug therapy to prevent neurologic sequelae (*24*). Several survivors (postal workers) in 2001 received corticosteroids for nonanthrax indications (*3*). On the basis of available evidence and absence of apparent side effects unique to treatment for systemic anthrax, adjunctive corticosteroids should be considered in patients who had a history of use of endocrine or corticosteroid therapy; edema, especially of the head or neck; evidence of anthrax meningitis; or vasopressor-resistant shock (*16*,*25*).

### Procedures and Surgical Interventions

High LF concentrations have been detected in pleural fluid (*4*) and in ascites (A. Boyer, unpub. data) from patients with inhalation anthrax. In addition, pleural fluid drainage has been positively associated with survival in a large case series (*26*). Drainage of pleural fluid and ascites is believed to improve survival by reducing the toxin level and by decreasing mechanical lung compression. These data support the need for early and aggressive drainage of any clinically or radiographically apparent pleural effusions; chest tube drainage is recommended over thoracentesis because many effusions will require prolonged drainage. Thoracotomy or video-assisted thoracic surgery might be required to remove gelatinous or loculated collections. Ascites should also be drained and monitored for reaccumulation; continuous drainage might be required.

Surgery might be contraindicated or indicated, depending on the type of anthrax. Surgery for cutaneous anthrax can lead to dissemination and poor outcome. Surgery is contraindicated for acute disease, with the exception of tracheotomy for airway obstruction and surgical intervention for large or circumferential extremity lesions causing compartment syndrome. Surgery may be indicated for gastrointestinal anthrax to identify and address potentially fatal complications, such as bowel ischemia, necrosis, and perforation (*27*). For injection anthrax, surgery is used to obtain diagnostic specimens to differentiate the infection from necrotizing fasciitis and to remove the necrotic nidus of infection, which may be a toxin and spore reservoir. Surgery for injection anthrax should be more limited than that for necrotizing fasciitis, and resection should be performed only to healthy tissue. Compression of soft tissues can be released by incision, excision, or fasciotomy and might be required for treatment of compartment syndrome (*28*). Diathermy dissection is advised to optimize hemostasis.

## Antimicrobial Selection Considerations for Prevention and Treatment

The approach to prevention and treatment of anthrax differs from that for other bacterial infections. The production of toxin, potential for antimicrobial drug resistance, frequent occurrence of meningitis, and presence of latent spores must be taken into account when selecting postexposure prophylaxis (PEP) or a combination of antimicrobial drugs for treatment of anthrax. In addition, most data on which these antimicrobial drug options are based predate the availability of many of the antimicrobial drugs discussed and are drawn from limited animal studies.

Among patients with inhalation anthrax during 1900–2005, antimicrobial drug combination therapy was more likely to be curative than antimicrobial drug monotherapy (*26*). There is also a theoretical benefit for combined use of bactericidal and protein synthesis inhibitor agents. Bactericidal agents have immediate killing effect. However, the high rates of illness and death seen with anthrax are caused, in part, by *B. anthracis* exotoxin production. In vitro toxin production is inhibited earlier by protein synthesis inhibitors than by bactericidal agents (*29*); this characteristic is associated with clinical benefit in streptococcal toxic shock and clostridial sepsis. Combination antimicrobial drug regimens were used in all 8 survivors of inhalation anthrax during 2001–2012; five survivors were also treated with a protein synthesis inhibitor (*4*,*26**,**30*). Patients hospitalized for systemic anthrax should be immediately treated with a combination of broad-spectrum intravenous antimicrobial drug treatment pending confirmatory test results because any delay may prove fatal.

Naturally occurring *B. anthracis* has variable β-lactam resistance, particularly to cephalosporins (*31*). Thus, cephalosporins are contraindicated. In addition, multidrug resistance in naturally occurring *B. anthracis* infection has been reported (*32*) and can be induced in vitro (*33*). This finding increases concern that a *B. anthracis* strain could be resistant to ≥1 recommended antimicrobial drug options or that use of β-lactams for a strain that was originally β-lactam susceptible could lead to development of β-lactam-resistance during prophylaxis, if adherence to treatment is poor, as was observed among postal workers in 2001 (*34*). Another concern is that β-lactam use could induce resistance during treatment, especially with the high bacterial loads characteristic of systemic anthrax. Thus, penicillin-based antimicrobial drug use requires a high index of suspicion for emergence of resistance.

Meningitis and hemorrhagic brain parenchymal infection has been observed in ≤50% of human anthrax cases (*16*,*26*). Thus, meningitis must be considered in all cases of systemic anthrax, and antimicrobial drugs used to treat possible meningitis must have good penetration of the central nervous system (CNS).

The presence of a spore form of *B. anthracis* requires prolonged antimicrobial drug prophylaxis. Incubation periods ≤43 days have been observed in humans (*2*) and 58 days in NHPs (*35*), and viable spores have been detected in lungs of NHPs for ≤100 days after aerosol exposures (*36*). Thus, persons exposed to aerosolized *B. anthracis* are presumed to be at risk for inhalation anthrax from ungerminated spores retained in their lungs after the initial exposure, including patients treated for any form of anthrax who were exposed to aerosolized spores. In addition, if antimicrobial drug treatment is initiated soon after exposure, animal studies suggest the acquired immune response might be blunted and not be protective (*35*). This finding suggests the need to continue antimicrobial drug therapy for 60 days to clear germinating organisms.

## Anthrax Prevention

Well-timed and effective PEP can potentially save thousands of lives (Technical Appendix Table 2). PEP of asymptomatic persons should ideally start as soon as possible after exposure because its effectiveness decreases with delay in implementation. In 2009, the US Advisory Committee on Immunization Practices recommended 60 days of antimicrobial drug prophylaxis for immediate protection and a 3-dose series of Anthrax Vaccine Adsorbed (AVA) BioThrax (Emergent Biosolutions Inc., Rockville, MD, USA) for long-term protection after exposure to anthrax (*37*). To ensure adequate and continued protection, everyone exposed to aerosolized *B. anthracis* spores should receive a full 60 days of PEP antimicrobial drugs, whether they are unvaccinated, partially vaccinated, or fully vaccinated.

### Antimicrobial Drugs

Ciprofloxacin, levofloxacin, and doxycycline are FDA-approved for the antimicrobial drug portion of PEP for inhalation anthrax in adults ≥18 years of age. No safety data are available for levofloxacin use beyond 30 days; thus, oral ciprofloxacin and doxycycline are recommended as first-line antimicrobial drugs for PEP. Alternative antimicrobial drugs that might be used for PEP if first-line agents are not tolerated or are unavailable include levofloxacin and moxifloxacin; amoxicillin and penicillin VK if the isolate is penicillin susceptible; and clindamycin. The antimicrobial drug linezolid cannot be used for extended periods. Also, the risk for development of resistance must be kept in mind if using β-lactam drugs.

### Vaccine

Clinical trials in humans have demonstrated evidence of seroconversion after 3 doses of AVA. The vaccine should be administered subcutaneously at diagnosis and 2 and 4 weeks later (*37*). AVA is not FDA-approved for PEP and could be made available under an Investigational New Drug protocol or an Emergency Use Authorization in a declared emergency.

## Antimicrobial Treatment for Systemic Disease with Possible Meningitis

Empiric treatment for anthrax in which anthrax meningitis is suspected or cannot be ruled out should include ≥3 antimicrobial drugs with activity against *B. anthracis*; ≥1 drug should have bactericidal activity, ≥1 should be a protein synthesis inhibitor, and all should have good CNS penetration (Technical Appendix Table 3). Intravenous combination treatment for systemic anthrax with possible meningitis should be provided for ≥2 weeks or until the patient is clinically stable, whichever is longer. Given the high mortality rate associated with meningitis, some expert panelists favored 3 weeks of treatment for patients in whom meningitis could not be ruled out.

Intravenous ciprofloxacin is preferred as the primary bactericidal component in the treatment of systemic disease on the basis of efficacy in NHP infection models and recent use for anthrax cases. Levofloxacin and moxifloxacin are considered equivalent alternatives to ciprofloxacin. The fluoroquinolones have adequate CNS penetration (*16*) and there are no reports of natural resistance.

The carbapenem class of antimicrobial drugs is highly resistant to β-lactamases and provides good CNS penetration. Meropenem is preferred as the second antimicrobial drug in the combination antimicrobial drug regimen for anthrax meningitis. If meropenem is unavailable, doripenem and imipenem/cilastatin are considered equivalent alternatives. Imipenem/cilastatin is associated with increased seizure risk (*38*) and should be used with caution in patients with suspected meningitis. If the *B. anthracis* strain is susceptible to penicillin (MIC <0.125 µg/mL), penicillin G or ampicillin are acceptable alternatives to carbapenems.

At least 1 antimicrobial drug that inhibits protein synthesis should be used to reduce exotoxin production. Linezolid is preferred as the first-line protein synthesis inhibitor. It is preferred over clindamycin because it is likely to provide better CNS penetration (*39*), although randomized controlled trials on treatment for CNS infections with either agent are lacking. However, linezolid toxicity issues must be taken into consideration. Myelosuppression, peripheral and optic neuropathies, and serotonin syndrome have been reported in patients receiving linezolid (*40*). Linezolid should be used cautiously in patients with pre-existing myelosuppression. In patients receiving monoamine oxidase inhibitors or serotonin reuptake inhibitors, the benefit of linezolid treatment should be weighed against the risk for serotonin toxicity and an alternative should be considered. If patients experience visual impairment, prompt ophthalmic evaluation is recommended. If patients have contraindications to linezolid use or it is unavailable, clindamycin is an acceptable alternative. Rifampin, although not a protein synthesis inhibitor, has been widely used for its synergistic effect with a primary drug and could also be used in this capacity if linezolid or clindamycin are unavailable. The protein synthesis inhibitor chloramphenicol has good CNS penetration and has historically been used to successfully treat anthrax. Where available, it could be an acceptable alternative if linezolid, clindamycin, and rifampin are unavailable. Doxycycline should not be used if meningitis is suspected because it does not adequately penetrate the CNS.

## Antimicrobial Treatment for Systemic Disease If Meningitis Is Ruled Out

With the following 4 exceptions, antimicrobial drug options for patients with systemic anthrax are similar to those for patients with suspected meningitis or when meningitis cannot be ruled out (Technical Appendix Table 4). First, treatment should include ≥2 antimicrobial drugs with activity against *B. anthracis*; ≥1 should have bactericidal activity and ≥1 should be a protein synthesis inhibitor. Second, initial intravenous combination treatment should be given for ≥2 weeks or until the patient is clinically stable, whichever is longer. Third, if the *B. anthracis* strain is susceptible to penicillin, then penicillin G is considered equivalent to the fluoroquinolone options for primary bactericidal treatment. Fourth, treatment with antimicrobial drugs that have good CNS penetration is not a crucial factor. Thus, meropenem is recommended as an acceptable alternative option than as a first-line antimicrobial drug, and vancomycin is also an acceptable alternative. Clindamycin and linezolid are considered equivalent first-line choices for protein synthesis inhibitors. Doxycycline is added as an alternative protein synthesis inhibitor option if linezolid or clindamycin are contraindicated or unavailable.

## Follow–up Oral Treatment for Systemic Disease

Once patients with systemic illness who were exposed to aerosolized spores have completed initial combination treatment, they should be transitioned to single-agent oral treatment to prevent relapse from surviving *B. anthracis* spores. Antimicrobial drug options are the same as those for PEP (Technical Appendix Table 2).

## Treatment for Cutaneous Anthrax without Systemic Involvement

Uncomplicated cutaneous anthrax has been successfully treated with a single oral antimicrobial drug (Technical Appendix Table 5). Oral fluoroquinolones (ciprofloxacin, levofloxacin, and moxifloxacin) and doxycycline are equivalent first-line agents. Clindamycin is an alternative option if fluoroquinolones and doxycycline are contraindicated or unavailable. Given the long history of successful treatment of localized uncomplicated cutaneous anthrax with penicillin, amoxicillin and penicillin VK are also alternative therapeutic options if the isolate is known to be susceptible to penicillin. However, adequate dosages must be used because of the potential for development of drug resistance during treatment with subtherapeutic dosing.

Duration of treatment for localized or uncomplicated cutaneous disease depends on the *B. anthracis* exposure source. If naturally acquired (e.g., animals with anthrax, products such as hides from animals with anthrax), a 7–10-day course of antimicrobial drugs is sufficient. If bioterrorism-related exposure or an aerosol exposure is suspected, the recommendation is the same as that for PEP (Technical Appendix Table 2) because the patient is likely to have also inhaled spores.

## Antitoxins

In case series of patients with cutaneous anthrax who received no treatment during 1890–1907 or antiserum during 1903–1907 (Technical Appendix Table 6), 23.7% of the 26,567 untreated patients died. In contrast, 11.8% of the 305 patients who received antiserum died. Although there are far fewer data for patients with inhalation anthrax, in the pre-antibiotic era, there were 3 survivors among 6 patients treated with antiserum (*26*).

There are currently 2 antitoxins in the CDC Strategic National Stockpile: raxibacumab (GlaxoSmithKline, London, UK) and Anthrax Immune Globulin Intravenous (AIGIV) (Cangene Corporation, Winnipeg, Manitoba, Canada). Both antitoxins inhibit binding of PA to anthrax toxin receptors and translocation of the 2 primary toxins (LT and ET) into cells. Raxibacumab is a recombinant, fully humanized, IgG1λ monoclonal antibody. AIGIV is a human polyclonal antiserum made from plasma of persons immunized with AVA, which might have some direct effect on LF and EF.

### Raxibacumab

Raxibacumab increased survival in animal studies when administered as monotherapy: 44% of rabbits treated with 40 mg/kg survived compared with no survivors in the control group (p = 0.003), and 50% and 64% of NHPs treated with 20 and 40 mg/kg, respectively, survived, compared with no survivors in the control group (p<0.01 and p<0.001, respectively) (*41*). When raxibacumab was combined with antimicrobial drugs, 82% of rabbits given raxibacumab plus levofloxacin survived compared with 65% given levofloxacin alone (p = 0.0874).

Raxibacumab appeared safe and well tolerated in 333 healthy adults who received the recommended dose of 40 mg/kg. Most adverse events were transient and mild to moderate in severity. Pruritis was noted in 2.1% of persons treated with raxibacumab and in none treated with placebo (*41*). Although raxibacumab has not been given to patients with systemic anthrax, it is FDA-approved for PEP and treatment for anthrax under the Animal Rule Summary (www.fda.gov/downloads/AdvisoryCommittees/.../UCM239734.ppt*).‎*

### Anthrax Immune Globulin

In animal studies, AIGIV increased survival when administered without an antimicrobial drug. Twenty-six percent of rabbits that received 15 U/kg survived, compared with 2% in the control group (p<0.001); 44% of NHPs that received the same dose survived, compared with 6% in the control group (p<0.05). Seventy-one percent of NHPs survived when 30 U/kg of AIGIV was used (p<0.001 compared with placebo). When AIGIV was added to antimicrobial drugs, 70% of rabbits treated with AIGIV plus levofloxacin survived, compared with 25% of control animals treated with intravenous immunoglobulin plus levofloxacin (p = 0.13).

AIGIV was evaluated in 74 healthy adult volunteers and appears safe and well tolerated at all doses tested. The most frequently reported adverse events were headache, pharyngolaryngeal pain, and nausea. A total of 19 patients with anthrax have received AIGIV as an adjunct to antimicrobial drugs: 3 had inhalation anthrax, 15 had injection anthrax, and 1 had gastrointestinal anthrax. All patients appeared to tolerate the antitoxin, and 13 survived: 2 with inhalation anthrax (*4**,**30*), 10 with injection anthrax (W.A. Bower, unpub. data), and 1 with gastrointestinal anthrax (*5*). AIGIV is not FDA approved and could be made available under an Investigational New Drug protocol or an Emergency Use Authorization during a declared emergency.

### Antitoxin Summary

Pre-antimicrobial era observational data for humans and animal studies indicate that anthrax antitoxins are effective when given without and with antimicrobial drugs. However antimicrobial drugs alone can also be effective if given early in the course of disease. During the 2001 outbreak, all 6 patients with inhalation anthrax who received intravenous antimicrobial drugs during the prodromal phase of their illness survived, and all 5 who received antimicrobial drugs after the prodromal phase died (*3*,*15*). Although anthrax antitoxin has a role in treatment for systemic anthrax, data on the optimal time to initiate it are lacking, and the opinions of expert panelists were mixed on this issue. Given that systemic anthrax has a high case-fatality rate and the risk for antitoxin treatment appears to be low, the potential benefit achieved by adding antitoxin to combination antimicrobial drug treatment outweighs the potential risk. An antitoxin should be added to combination antimicrobial drug treatment for any patient for whom there is a high level of clinical suspicion for systemic anthrax. Although there is some experience with AIGIV use in humans, there are no major medical, operational, or logistical considerations that clearly favor use of 1 antitoxin over another in adults with systemic anthrax.

## Conclusions

Biodefense experts currently place *B. anthracis* at or near the top of the list for potential threat agents: it is available and easy to disseminate, and systemic anthrax infection has a high mortality rate. Before 2001, mortality rates for patients with inhalation anthrax approached 90%. Since that time, 8 (53%) of 15 known patients with inhalation anthrax have survived. These survivors were given an early diagnosis, began combination antimicrobial drug treatment to eradicate the bacteria and inhibit toxin production, and had aggressive pleural effusion management. The principles of prevention and clinical care provided in this report are based on currently available data and the lessons learned from recent anthrax events.

There are many opportunities for improving prevention of and treatment for anthrax. Some evidence suggests the duration of PEP antimicrobial drug use could be decreased when given in conjunction with AVA. Further research could provide sufficient evidence to decrease the length of the antimicrobial drug component of PEP. Efforts are ongoing to develop point-of-care assays. Assays that quantify toxin levels or antibody response could enable earlier diagnosis and treatment and help identify optimal timing for antitoxin administration. Pharmacokinetic data for antimicrobial drugs used to treat anthrax are limited and almost no pharmacokinetic data for CNS are available, but such data are needed for dosing recommendations. Animal studies are needed to evaluate synergistic or antagonistic activity in multiple antimicrobial drug combinations; to study the survival effect of corticosteroids or other adjunctive treatments for meningitis, edema, and vasopressor-resistant shock; and to determine whether currently recommended antitoxin dosing is optimal. Currently available antitoxins counteract extracellular toxins. Studies are needed to assess whether intracellular toxin inhibitors, some of which are FDA approved for other indications, are efficacious in animal models of anthrax infection. Such agents might be of use in patients in the fulminant phase of disease. When research needed to change the anthrax mortality rate is conducted, *B. anthracis* will become a less competent, and therefore less desirable, bioweapon.

Technical Appendix Diagnosis, monitoring and assessment, therapy, prophylaxis, and mortality rates of patients with anthrax.
