# Supplementary material for: Centers for Disease Control and Prevention Expert Panel Meetings on Prevention and Treatment of Anthrax in Adults
Source: Emerg Infect Dis. 2014 Feb;20(2):e130687. doi: 10.3201/eid2002.130687 (PMC3901462; doi:10.3201/eid2002.130687)
Supplement: Technical Appendix — Diagnosis, monitoring and assessment, therapy, prophylaxis, and mortality rates of patients with anthrax. [file 13-0687-Techapp-s1.pdf]

Article DOI: http://dx.doi.og/10.3201/eid2002.130687

# Centers for Disease Control and Prevention Expert Panel Meetings on Prevention and Treatment of Anthrax in Adults

## Technical Appendix

## Diagnosis, monitoring and assessment, therapy, prophylaxis, and mortality rates of patients with anthrax

Technical Appendix Table 1. Diagnostic assessment and monitoring of adults with systemic anthrax*

| | Unique findings for systemic anthrax | |
|---|---|---|
| Test | Initial | Serial monitoring |
| Complete blood count | Marked hemoconcentration; thrombocytopenia may not be present; leukocyte count frequently at reference level | Anemia can suddenly develop; thrombocytopenia onset often associated with hemolytic anemia; leukocytosis usually not seen until late in disease |
| Electrolytes, renal panel, lactate level | Decreased sodium level; $HCO_3$ level can be at reference level even with sepsis; increased blood urea nitrogen level | |
| Liver enzymes, serum albumin | Mildly elevated transaminase levels; hypoalbuminemia related to acute infection | |
| PT, PTT, D-dimer, fibrinogen | Reference PT/PTT at admission does not exclude coagulopathy or disseminated intravascular coagulopathy | Low threshold for hypercoagulability workup: including haptoglobin, lactate dehydrogenase, fibrin split products. If evidence of hemolytic anemia, assess ADAMTS 13 (von Willebrand factor–cleaving protease) |
| Erythrocyte sedimentation rate, CRP | Useful for characterizing inflammatory response. Low CRP characteristic in injection anthrax | |
| Gram stain, cultures, toxin assays | Initial testing on any accessible fluid: blood, serum, cerebrospinal and pleural fluid, ascites, wound exudates, bronchial aspirates | Cultures usually negative after antimicrobials, but toxin may be detectable at multiple time points |
| Cardiac enzymes with or without B-type natriuretic peptide | Troponin leak caused by increased cardiac demands from acute infection (especially if atrial fibrillation with rapid ventricular response) | |
| Electrocardiogram/continuous telemetry | Atrial fibrillation with rapid ventricular response | |
| Posterior–anterior and lateral chest radiograph | Any abnormality: characteristic mediastinal widening and pleural effusions may be subtle or inapparent | Daily chest radiographs or other thoracic imaging until pleural effusions are stable or decreasing |

| | Unique findings for systemic anthrax | |
|---|---|---|
| Test | Initial | Serial monitoring |
| Chest computerized tomography | Evaluate for severity of pleural effusions, presence of mediastinal widening or pericardial effusion, and to rule out thromboembolic disease | Repeat if major clinical status change |
| Lumbar puncture | Perform at admission unless contraindicated | Perform for headache/confusion or other neurologic symptoms; meningeal signs are usually not present until late stage if meningitis is present |
| Other imaging | As relevant to site of exposure; to evaluate edema, inflammation and necrosis | Perform for headache/confusion or other neurologic symptoms; meningeal signs are usually not present until late stage if meningitis is present |
| Echocardiogram | Evaluate for pericardial effusion and myocardial dysfunction | |

*Medical history, physical examination, and vital signs with pulse oximetry at admission should be followed up by regular careful monitoring. This analysis applies even in patients who appear clinically improved because of the potential for sudden decompensation. Although standard blood work and diagnostic tests are recommended to evaluate acute infectious illnesses, anthrax is associated with unique findings. PT, prothrombin time; PTT, partial thromboplastin time; ADAMTS 13, a disintegrin and metalloproteinase with a thrombospondin type 1 motif, member 13; CRP, C-reactive protein.

Technical Appendix Table 2. Oral postexposure prophylaxis for infection with *Bacillus anthracis**

| Treatment |
|---|
| For all strains, regardless of penicillin susceptibility or if susceptibility is unknown |
| **Ciprofloxacin, 500 mg every 12 h** |
| OR |
| **Doxycycline, 100 mg every 12 h** |
| OR |
| Levofloxacin, 750 mg every 24 h |
| OR |
| Moxifloxacin, 400 mg every 24 h |
| OR |
| Clindamycin, 600 mg every 8 h† |
| OR |
| Alternatives for penicillin-susceptible strains |
| Amoxicillin, 1 g every 8 h |
| OR |
| Penicillin VK, 500 mg every 6 h |

*Preferred drugs are indicated in **boldface**. Alternative drugs are listed in order of preference for treatment for patients who cannot take first-line treatment or if first-line treatment is unavailable.
†Based on in vitro susceptibility data rather than studies of clinical efficacy.

Technical Appendix Table 3. Intravenous treatment for systemic anthrax with possible/confirmed meningitis*

| Treatment |
|---|
| Bactericidal agent (fluoroquinolone) |
| **Ciprofloxacin, 400 mg every 8 h** |
| OR |
| Levofloxacin, 750 mg every 24 h |
| OR |
| Moxifloxacin, 400 mg every 24 h |
| PLUS |
| Bactericidal agent (β-lactam) |
| For all strains, regardless of penicillin susceptibility or if susceptibility is unknown |
| **Meropenem, 2 g every 8 h** |
| OR |
| Imipenem, 1 g every 6 h† |
| OR |
| Doripenem, 500 mg every 8 h |
| OR |
| Alternatives for penicillin-susceptible strains |
| Penicillin G, 4 million units every 4 h |
| OR |
| Ampicillin, 3 g every 6 h |
| PLUS |
| Protein synthesis inhibitor |
| **Linezolid, 600 mg every 12 h‡** |
| OR |
| Clindamycin, 900 mg every 8 h |
| OR |
| Rifampin, 600 mg every 12 h§ |
| OR |
| Chloramphenicol, 1 g every 6–8 h¶ |

*Duration of treatment: ≥2–3 weeks until clinical criteria for stability are met (see text). Patients exposed to aerosolized spores will require prophylaxis to complete an antimicrobial drug course of 60 d from onset of illness (see Technical Appendix Table 2 [postexposure prophylaxis]). Systemic anthrax includes anthrax meningitis; inhalation, injection, and gastrointestinal anthrax; and cutaneous anthrax with systemic involvement, extensive edema, or lesions of the head or neck. Preferred drugs are indicated in **boldface**. Alternative drugs are listed in order of preference for treatment for patients who cannot take first-line treatment, or if first-line treatment is unavailable.
†Increased risk for seizures associated with imipenem/cilastatin treatment.
‡Linezolid should be used with caution in patients with thrombocytopenia because it might exacerbate it. Linezolid use for >14 d has additional hematopoietic toxicity.
§Rifampin is not a protein synthesis inhibitor. However, it may be used in combination with other antimicrobial drugs on the basis of its in vitro synergy.
¶Should only be used if other options are not available because of toxicity concerns.

Technical Appendix Table 4. Intravenous therapy for systemic anthrax when meningitis has been excluded*

| Bactericidal drug |
| --- |
| For all strains, regardless of penicillin susceptibility or if susceptibility is unknown |
| **Ciprofloxacin, 400 mg every 8 h** |
| OR |
| Levofloxacin, 750 mg every 24 h |
| OR |
| Moxifloxacin, 400 mg every 24 h |
| OR |
| Meropenem, 2 g every 8 h |
| OR |
| Imipenem, 1 g every 6 h† |
| OR |
| Doripenem, 500 mg every 8 h |
| OR |
| Vancomycin, 60 mg/kg/d intravenous divided every 8 h (maintain serum trough concentrations of 15–20 μg/mL) |
| OR |
| Alternatives for penicillin-susceptible strains |
| Penicillin G, 4 million units every 4 h |
| OR |
| Ampicillin, 3 g every 6 h |
| PLUS |
| Protein synthesis inhibitor |
| **Clindamycin, 900 mg every 8 h** |
| OR |
| **Linezolid, 600 mg every 12 h‡** |
| OR |
| Doxycycline, 200 mg initially, then 100 mg every 12 h§ |
| OR |
| Rifampin, 600 mg every 12h¶ |

*Duration of treatment: for 2 weeks until clinical criteria for stability are met (see text). Patients exposed to aerosolized spores will require prophylaxis to complete an antimicrobial drug course of 60 d from onset of illness (see Technical Appendix Table 2 [postexposure prophylaxis]). Systemic anthrax includes anthrax meningitis; inhalation, injection, and gastrointestinal anthrax; and cutaneous anthrax with systemic involvement, extensive edema, or lesions of the head or neck. Preferred drugs are indicated in **boldface**. Alternative drugs are listed in order of preference for treatment for patients who cannot take first-line treatment, or if first-line treatment is unavailable.
†Increased risk for seizures associated with imipenem/cilastatin treatment.
‡Linezolid should be used with caution in patients with thrombocytopenia because it might exacerbate it. Linezolid use for >14 d has additional hematopoietic toxicity.
§A single 10–14 d course of doxycycline is not routinely associated with tooth staining.
¶Rifampin is not a protein synthesis inhibitor. However, it may be used in combination with other antimicrobials drugs on the basis of its in vitro synergy.

Technical Appendix Table 5. Oral treatment for cutaneous anthrax without systemic involvement*

| Treatment |
| --- |
| For all strains, regardless of penicillin susceptibility or if susceptibility is unknown |
| **Ciprofloxacin, 500 mg every 12 h** |
| OR |
| **Doxycycline, 100 mg every 12 h** |
| OR |
| **Levofloxacin, 750 mg every 24 h** |
| OR |
| **Moxifloxacin, 400 mg every 24 h** |
| OR |
| Clindamycin, 600 mg every 8 h† |
| OR |
| Alternatives for penicillin-susceptible strains |
| Amoxicillin, 1 g every 8 h |
| OR |
| Penicillin VK, 500 mg every 6 h |

*Preferred drugs are indicated in **boldface**. Alternative drugs are listed in order of preference for treatment for patients who cannot take first-line treatment, or if first-line treatment is unavailable. Duration of treatment is 60 d for bioterrorism-related cases and 7–10 d for naturally acquired cases.
†Based on in vitro susceptibility data, rather than studies of clinical efficacy.

Technical Appendix Table 6. Mortality rates for untreated and serum-treated cutaneous anthrax*

| Location (reference) | Years | No. cases | No. deaths | Mortality rate, % |
|---|---|---|---|---|
| Untreated cutaneous anthrax | | | | |
| Massachusetts, USA (*1*) | 1888–1919 | 141 | 51 | 36.2 |
| Italy (*2,3*) | 1890–1900 | 24,052 | 5,812 | 24.2 |
| Great Britain (*4–6*) | 1899–1907 | 216 | 52 | 24.1 |
| Germany (*7,8*) | 1910–1923 | 1,798 | 281 | 15.6 |
| Schleswig-Holstein, Germany (*1*) | 1910–1925 | 176 | 31 | 17.6 |
| Hamburg, Germany (*1*) | 1910–1925 | 150 | 58 | 38.7 |
| New York, NY, USA (*1*) | 1919–1920 | 34 | 11 | 32.4 |
| Total | NA | 26,567 | 6,296 | 23.7 |
| Serum-treated cutaneous anthrax | | | | |
| Italy *(2,3*) | 1903 | 164 | 10 | 6.1 |
| Germany (*9*) | 1904 | 1,048 | 44 | 4.2 |
| Great Britain (*4,5,10*) | 1904–1907 | 85 | 24 | 28.2 |
| Italy (*10*) | 1905 | 56 | 2 | 3.6 |
| Uruguay (*11*) | 1915–1923 | 309 | 58 | 18.8 |
| Argentina (*12*) | 1917 | 415 | 18 | 4.3 |
| Italy (*1*) | 1920 | 160 | 10 | 6.3 |
| United States (*13*) | 1920–1923 | 16 | 3 | 18.7 |
| Argentina (*14–16*) | 1922 | 15 | 3 | 20.0 |
| United States (*17*) | 1932–1941 | 19 | 0 | 0.0 |
| United States (*18*) | 1933–1939 | 21 | 1 | 4.8 |
| Great Britain (*19*) | 1941 | 52 | 6 | 11.5 |
| Total | NA | 2,360 | 179 | 7.6 |

*NA, not applicable.

## References


1. Canright CM. Human anthrax and its treatment. A report of three cases [in Chinese]. Chinese Medical Journal. 1928;42:479–96.

2. Sclavo A. Present status of anticarbonchiosa serum therapy [in Italian]. Revista d’Igiene e Sanità Pubblica. 1903; 14:519–587.

3. Sclavo A. Serum treatment of anthrax in man [in Italian]. Rivista Italiana Igiene. 1954;14:161–75.

4. Page CH. British industrial anthrax: part I. J Hyg (Lond). 1909;9:279–315. http://dx.doi.org/10.1017/S0022172400016338

5. Page CH. British industrial anthrax: part II. J Hyg (Lond). 1909;9:357–98. http://dx.doi.org/10.1017/S0022172400016405

6. Legge TM. Industrial anthrax: lecture I. Br Med J. 1905;1:529–31. http://dx.doi.org/10.1136/bmj.1.2306.529

7. Graf P. A lot about anthrax in the past 25 years, 1926 [in German]. Munchener Medizinische Wochenschrift. 1888; 35:73.

8. Graf P. To the clinic and treatment of meschlischen anthrax after experiences of Stadtur Jen House Neumunster in the past 25 years [in German]. Deutsche Zeitschrift fur Chirurgie. 1926;516:198–9.

9. Mendez J. Anthrax antitoxin [in German]. Centralbl Bakteriologie Orig I. 1904;37:405–10.

10. Legge TM. Industrial anthrax: lecture II. Br Med J. 1905;1:589–93. http://dx.doi.org/10.1136/bmj.1.2307.589

11. Prat D. Treatment of carbuncle [in Spanish]. Anales de la Facultad de Medicina, Montevideo. 1923;8:576.

12. Penna J, Cuenca JB, Kraus R. Normal serum human anthrax treatment [in Spanish]. Prensa Médica Argentina. 1917;3:261–99.

13. Santee HE. Anthrax and its treatment. Ann Surg. 1923;78:326–31. http://dx.doi.org/10.1097/00000658-192309000-00003

14. Vaccarezza RF, Inda FF, Posse R. Human anthrax treatment: normal human serum and general results of peptoma (part 1) [in Spanish]. La Semana Médica. 1922;22:943–69.

15. Vaccarezza RF, Inda FF, Posse R. Human anthrax treatment: normal human serum and general results of peptoma (part 2) [in Spanish]. La Semana Médica. 1922;24:1053–69.

16. Vaccarezza RF, Inda FF, Posse R. Human anthrax treatment: normal human serum and general results of peptoma (part 3) [in Spanish]. La Semana Médica. 1922;29:865–907.

17. Lucchesi PF, Gildersleeve N. The treatment of anthrax. JAMA. 1941;116:1506–8. http://dx.doi.org/10.1001/jama.1941.02820140018005

18. Gold H. Anthrax. A review of sixty cases, with a report on the therapeutic use of sulfonamide compounds. Arch Intern Med. 1942;70:785–821. http://dx.doi.org/10.1001/archinte.1942.00200230098008

19. Hodgson AE. Cutaneous anthrax. Lancet. 1941;237:811–3. http://dx.doi.org/10.1016/S0140-6736(00)61181-9
